# A prismatic view of the epigenetic-metabolic regulatory axis in breast cancer therapy resistance

**DOI:** 10.1038/s41388-024-03054-9

**Published:** 2024-05-08

**Authors:** Chandrima Das, Apoorva Bhattacharya, Swagata Adhikari, Atanu Mondal, Payel Mondal, Santanu Adhikary, Siddhartha Roy, Kenneth Ramos, Kamlesh K. Yadav, John A. Tainer, Tej K. Pandita

**Affiliations:** 1 Biophysics and Structural Genomics Division, Saha Institute of Nuclear Physics, 1/AF Bidhannagar, Kolkata, 700064 India; 2 Homi Bhabha National Institute, Mumbai, 400094 India; 3 Structural Biology and Bioinformatics Division, Council of Scientific and Industrial Research (CSIR)-Indian Institute of Chemical Biology, Kolkata, 700032 India; 4 Center for Genomics and Precision Medicine, Texas A&M University, School of Medicine, Houston, TX 77030 USA; 5 School of Engineering Medicine, Texas A&M University, School of Medicine, Houston, TX 77030 USA; 6 The University of Texas MD Anderson Cancer Center, Houston, TX 77030 USA

**Keywords:** Biochemistry, Cell biology

## Abstract

Epigenetic regulation established during development to maintain patterns of transcriptional expression and silencing for metabolism and other fundamental cell processes can be reprogrammed in cancer, providing a molecular mechanism for persistent alterations in phenotype. Metabolic deregulation and reprogramming are thus an emerging hallmark of cancer with opportunities for molecular classification as a critical preliminary step for precision therapeutic intervention. Yet, acquisition of therapy resistance against most conventional treatment regimens coupled with tumor relapse, continue to pose unsolved problems for precision healthcare, as exemplified in breast cancer where existing data informs both cancer genotype and phenotype. Furthermore, epigenetic reprograming of the metabolic milieu of cancer cells is among the most crucial determinants of therapeutic resistance and cancer relapse. Importantly, subtype-specific epigenetic-metabolic interplay profoundly affects malignant transformation, resistance to chemotherapy, and response to targeted therapies. In this review, we therefore prismatically dissect interconnected epigenetic and metabolic regulatory pathways and then integrate them into an observable cancer metabolism-therapy-resistance axis that may inform clinical intervention. Optimally coupling genome-wide analysis with an understanding of metabolic elements, epigenetic reprogramming, and their integration by metabolic profiling may decode missing molecular mechanisms at the level of individual tumors. The proposed approach of linking metabolic biochemistry back to genotype, epigenetics, and phenotype for specific tumors and their microenvironment may thus enable successful mechanistic targeting of epigenetic modifiers and oncometabolites despite tumor metabolic heterogeneity.

## Introduction

Cell metabolism comprises a network of interconnected pathways that ultimately provide the essential biomolecules required for cell survival and subsequent physiological functions. These pathways therefore can promote cancer cell survival, phenotypic transformations, and development of drug resistance. Large-scale integrative metabolomics analysis has identified tightly regulated biochemical pathways and new metabolic targets associating with specific phenotypes [1] (https://www.metabolicatlas.org/). Critically these pathways provide energy in the form of ATP as well as precursors for biomass production; they therefore are integral to the molecular sub-classification of tumors and cellular health. Moreover, epigenetics provides master keys to deregulation and reprogramming for the adaptive metabolic pathways that enable tumor survival during progression and development of drug resistance. Strikingly, mutations of various epigenetic regulators have been identified in about 50% of human cancers, and tumors without such mutations have expression changes coinciding with altered epigenetic activity [2]. Thus, investigating the epigenetic-metabolite axis and its association with tumor molecular classifications may hold promise for the development of advanced strategies targeting enzyme reactions and their pathway networks.

Molecular classification of breast tumors has played a vital and exemplary role in tailoring cancer treatment strategy and improving patient survival outcomes. Traditionally, different subtypes were classified based on stage and histopathology grade, molecular marker expression, and genetic diversity information. Breast cancer is thus broadly classified into four groups: Luminal A, Luminal B, ErbB2/HER2 (Erb-B2 Receptor Tyrosine Kinase 2/ human epidermal growth factor receptor 2) positive, and basal-like. Luminal A includes tumors that are estrogen receptor (ER) positive and progesterone receptor (PR) positive, but negative for HER2. These cancers are likely to benefit from hormone therapy. Luminal B includes tumors that are ER positive, PR negative, and HER2 positive. These cancers may benefit from hormone therapy and treatment targeted to HER2. Group 3 includes tumors that are ER/PR negative, but ErbB2/ HER2-positive subtype and show ERBb2 gene amplification. The basal-like breast cancers are generally ER/PR/HER2 negative and show pronounced expression of Cytokeratin 5/6 [3, 4]. Triple-negative breast cancer (TNBC), a major subclass of basal-like breast carcinoma (almost 70%), is one of the most aggressive forms of breast cancer [5].

In making therapeutic decisions, the Gallen 2013 consensus guideline [6] supported association of subtypes with clinical features, which often depend upon metabolic deregulation and reprogramming. These include but are not limited to size, proliferation rate, histological grade, and node invasion. For luminal A and B subtypes, endocrine therapy is the most commonly used therapeutic regimen, with the added use of chemotherapeutic drugs in most cases of luminal B. The major treatment strategy for the ErbB2/HER2 positive subtype of breast cancer is anti-HER2 drugs along with chemotherapy [7]. Due to the absence of endocrine receptors and HER2 expression, the mainstay treatment strategy for TNBC is chemotherapy [8]. However, novel combinatorial therapeutic strategies are being employed to treat advanced cancers. For instance, combination therapies using PARP-inhibitors with carboplatin are being used for patients harboring BRCA1/2 mutation [9], whereas a combination of immunotherapy with chemotherapy is utilized for treating PDL-1 positive TNBC patients which have undergone metastasis [10].

Fundamentally, energy metabolism reprogramming enables the development of resistance towards chemotherapy, hormone therapy, and HER2-targeted therapy [11–14]. Furthermore, a metabolic symbiosis is maintained between cancer cells and their surrounding microenvironment at primary and metastatic sites of tumor [15, 16]. Critically, this symbiosis fuels the high oncogenic biosynthetic and bioenergetic demand of the growing tumor, which can then mediate chemoresistance [17, 18].

## The reprogrammed metabolic landscape of breast cancer

Metabolic rewiring is a hallmark of cancer [19]. Otto Warburg first reported that in cancer cells, glycolysis predominates under aerobic conditions, while in normal cells, mitochondrial metabolism is favored in the presence of oxygen [20, 21]. While switching to glycolysis lowers efficient ATP production, in cancer cells this is compensated by accelerated cellular intake of glucose and a fast supply of ATP as energy demands increase due to enhanced cell proliferation rates during tumorigenesis. Furthermore, glycolytic intermediates allow divergence of different macromolecule biosynthetic pathways including amino acid and nucleotide biosynthesis to support the high proliferation rate of cancer cells. The glycolytic phenotype of cancer cells is acquired by various means including overexpression of glucose transporters and glycolytic enzymes that are regulated by oncogenic activation. For example, glucose transporter GLUT1 and glucose metabolic enzyme lactate dehydrogenase A (LDH-A) has been observed to be upregulated in various cancer types including breast cancer and observed to be associated with cancer drug resistance and metastasis [22, 23]. Another glucose-transported GLUT3 has been reported to be upregulated in breast cancer due to the overexpression of LIM protein Ajuba and promotes glucose uptake and chemoresistance in TAZ-GLUT3/Survivin mediated pathway [24]. Amino acid metabolism, among the three interconnecting central metabolic axes (carbohydrate, amino acid and lipid), also plays a crucial role in therapeutic intervention [25]. Similarly, upregulation of lipid and sterol metabolism has also been studied to be upregulated in different breast cancer subtypes [26].

Pavlova and Thompson introduced six emerging hallmarks of cancer metabolism to signal treatment options based on metabolic alterations [27]. These are (1) Altered amino acid and glucose uptake; (2) Opportunistic mode of nutrient procurement; (3) Use of glycolysis and tricarboxylic acid (TCA) cycle intermediates; (4) Augmented demands for Nitrogen; (5) Metabolic interactions with the tumor microenvironment; and 6) Modifications in metabolite driven gene regulation.

Glycolysis, TCA, and oxidative phosphorylation (OXPHOS) are the fundamental carbohydrate metabolic pathways that co-orchestrate biosynthetic pathways, and energy production. In cancer, metabolic rewiring shifts cellular energetics more towards glycolysis or OXPHOS or sometimes acquire glycolysis/OXPHOS hybrid metabolic phenotype in response to intrinsic and extrinsic cues [28].

Apart from being the building block of proteins, intermediates of amino acid metabolic pathways are essential in maintaining the redox balance of cells and epigenetic regulation of proto-oncogene, oncogene, and tumor suppressor genes [29]. Upon therapeutic intervention, the cancer cells adapt themselves in such a way that they can overcome the toxic effect of chemotherapeutic drugs by altering their metabolic landscape to suppress cell death, promote cell proliferation, activate drug efflux pumps and ultimately become resistant against specific class of drugs [30–32].

Altered lipid metabolism is a defence mechanism of cancer cells to develop resistance against chemotherapeutic treatment. Fatty acid can act as an energy source to support cell proliferation and cell survivability.

Therefore, cancer cells use metabolic plasticity as a strategy to bypass therapy. So, different metabolic adaptations offer clues to the acquisition of therapy resistance in different subtypes of breast cancer.

## Metabolic architecture in different breast cancer subtypes

During tumor emergence, the epigenetic regulated expression of metabolic enzymes control the metabolite pool size that ultimately contributes to aggressiveness [33]. This metabolic reprogramming differentially regulates the etiology of different breast cancer subtypes. Yet much of the subtype specificity remains to be defined as bulk tumor metabolome may not reflect intrinsic tumor cell changes in metabolism. Emerging spatial metabolomics from high-resolution and single-cell metabolomics technologies will increasingly reveal the nature of tumor subtype-specific metabolomic adaptations. Here we provide specific exemplary enzyme-metabolite associations below.

### ER-α positive/ HER2 negative

In ER-positive/HER2-negative breast cancer, phosphatidylinositol-4,5-bisphosphate 3-kinase catalytic subunit alpha (PIK3CA) mutational status correlates with high glucose uptake and glycolysis dependence [34]. Notably, estrogens, such as estradiol (E2), which is the strongest stimulator in estrogen receptor (ER)-positive breast cancer, increase expression of insulin receptors that facilitate glucose uptake while reducing lipase activity in the adipose tissues [35]. In response to high glucose, insulin signaling initiates secretion of insulin-like growth factor 1 (IGF-1), which binds to its cognate IGF-1 receptor (IGF-1R). This process activates both phosphoinositide 3 kinase/Akt/mammalian target of rapamycin (PI3K/Akt/mTOR) and mitogen-activated protein kinase/ extracellular signal-regulated kinase (MAPK/ERK) downstream signaling pathways and promotes cell proliferation [36]. Among these signaling cascades, mTOR has a major role in tumorigenesis when abnormally expressed in cancer cells, as it influences multiple signaling networks, impacting apoptosis, growth, and autophagy [37]. Activation of mTOR effects the function of 40S ribosomal S6 kinase 1 (S6K1), which ultimately phosphorylates estrogen receptor alpha (ER-α) on serine 167 leading to transcriptional activation of estrogen-responsive genes [38]. IGF1 and its receptor IGF-1R are also estrogen-signaling targets that operate autocrine signaling in breast cancer cells. Thus, the combination of estrogen and insulin signaling mediate the growth and proliferation of luminal breast cancer by enhancing the glycolytic pathway.

Glutamine metabolism is positively correlated with advancement of ER-positive breast cancer and associated with endocrine resistance [39]. Cappelletti et al. reported that the luminal B subtype of breast cancer largely depends on fatty acid metabolism to meet energy demands [40]. Furthermore, luminal type breast cancer cells can switch between glycolysis and mitochondrial metabolism depending upon glucose availability [41]. With high glucose availability, estrogen upregulates glycolysis and suppresses the TCA cycle, whereas, under glucose deprivation conditions, it inhibits glycolysis and promotes the TCA cycle by activating pyruvate dehydrogenase (PDH) to ensure cellular survival [41].

### HER2-Positive

HER2 amplification and its phosphorylation activates the PI3K/AKT signaling pathway, which is known to regulate glucose transporter 4 (GLUT4) expression, thereby promoting glucose uptake and glycolysis in HER2-positive early-stage breast cancer [42, 43]. Additionally, HER2 signaling induces 6-phosphofructo-2-kinase/fructose-2,6-biphosphatase 3 (PFKFBP3) expression which augments glycolysis, and is associated with trastuzumab resistance [44]. Tumor cells overexpressing HER2 show marked increases in lactate dehydrogenase-A (LDHA) resulting in high intra-tumor lactate production to sustain glycolysis and cancer cell growth [45, 46].

High levels of truncated isoform of dopamine and c-AMP regulated phosphoprotein (t-DARPP) are frequently seen in HER2-positive breast cancer which activate IGF-1R signaling through heterodimerization of HER2 and IGF-1R [47]. ErBb2 in association with mitochondrial heat shock protein 70 (HSP70) can translocate into mitochondria to negatively regulate the functions of complexes I, II, and IV of the mitochondrial electron transport chain (ETC), to alter OXPHOS.

HER2-positive breast cancer cells display higher levels of glutamine and fatty acid metabolism [48, 49]. Lipogenic enzyme expression increases at the transcriptional level [50], and fatty acid synthase (FASN) as well as acetyl-CoA carboxylase alpha (ACCα) at the translational level are positively correlated with HER2 oncogenic amplification [51]. Global metabolic profiling and multi-omics network approaches have shown that exogenous palmitate inhibits fatty acid synthesis to eventually feed back into glycolysis and amino acid metabolic pathways, creating a lipotoxicity in HER2-positive SKBR3 cells [52]. Additionally, high expression of enzymes involved in glutamine metabolism, including glutaminase (GLS1), glutamine dehydrogenase (GDH) and the glutamine transporter viz. alanine, serine, cysteine transporter type 2 (ASCT2), in HER2-positive breast cancer suggest dependence on active glutamine metabolism [53].

### Triple negative breast cancer (TNBC)

TNBC cells show increased reliance on glycolysis, altered glucose, fatty acid, and amino acid metabolism, which contributes to the increased cellular bioenergetic demands as the cancer continues to proliferate and metastasize [17]. Interestingly, receptor tyrosine kinase- Epidermal growth factor receptor (EGFR) and Mesenchymal Epithelial Transition (MET) signaling are intricately related to metabolic alterations in TNBC [54]. Moreover, TNBC displays excessive uptake of glucose and lactose due to high glucose transporter (GLUT) and monocarboxylate transporter (MCT) expression on its plasma membrane. The oncoprotein c-MYC represses the transcription of TXNIP (Thioredoxin interacting protein), a potent repressor of glycolysis [55], thus enhancing glycolytic rate in TNBC. The dependence of TNBC cells on anaerobic glycolysis is evident, as the heightened expression of LDHA and LDHB isozymes in patients is associated with poor clinical prognosis [56].

Conversely, the rate of gluconeogenesis is lowered in TNBC. For example, reduced expression of Fructose 1,6-Bisphosphatase 1 (FBP1), one of the three important rate-limiting enzymes of this pathway, has been reported in TNBCs but not in the luminal type of cancers. FBP1 expression is positively correlated with ER-positive breast cancer, which serves as a distinguishing mark between ER-positive and ER-negative subtypes [57]. Dong and his colleagues established that silencing of FBP1 is essential for Snail-mediated induction of epithelial to mesenchymal transition (EMT) and the development of cancer stem cell (CSC) like properties. This in turn imparts glycolytic advantages to the cells, thus reducing reactive oxygen species (ROS) generation and promoting resistance in TNBC [58]. Additionally, it has been reported that patients with upregulated essential amino acid metabolism were more prone to developing chemoresistance [59].

Along with FASN and ACCα, which were discussed above, recent studies have identified overexpression of acetyl-CoA synthetase 2 (ACSS2), an enzyme that converts acetate to acetyl-CoA, in response to low nutrient availability and hypoxia. Importantly, targeting ACSS2 can improve treatment response in TNBC [60]. Notably, the evolving role of non-acetyl acylation such as butyrylation is emerging as a new class of Histone modifications that can promote expression of gene involved in breast cancer progression [61].

## Metabolic adaptations during advanced metastatic stages

Metabolic heterogeneity in primary tumor cells dictates their metastatic potential and site-specific metastasis [62]. Compared to non-metastatic cancer cells, the enhanced metastatic potential of breast cancer cells is potentiated by both glycolysis and OXPHOS [63]. Breast cancer cell populations with enhanced OXPHOS are mediated by the peroxisome proliferator-activated receptor-gamma coactivator-1alpha (PGC1a) pathway to metastasize to bone and lung. Those with increased glycolysis through the hypoxia-inducible factor 1-apha/ 3-phosphoinositidine-dependent kinase (HIF1α/PDK1) network show liver metastasis [64, 65]. Protein expression profiling shows high glycolysis and oxidative metabolism along with the pentose phosphate pathway rendering certain growth advantages in breast cancer brain metastasis. Jinyu Chen et al. reported that fructose-1,6-bisphosphatase 2 (FBP2) expression is upregulated in brain metastasized cells of breast cancer origin to sustain and promote cancer cell survival by enhancing gluconeogenesis, as the absence of FBP2 compromises the proliferation and survival of these cells [66].

Metastatic cells show increased levels of reactive oxygen species, alterations in amino acid metabolism, and changes in ATP and the tricarboxylic acid (TCA) cycle that reflect metabolic adaptions [67, 68]. Additionally, aberrations in amino acid and lipid metabolism are associated with initiation, aggressiveness, and progression to metastasis as well as chemoresistance. The non-essential amino acid glutamine acts an important nutrient whose metabolism is likely a positive factor in promoting tumor metastasis. Increased glutamine production is brought about by metabolic reprogramming of the glutamine synthesis pathways [69]. Essential branched-chain amino acids (BCAAs) such as isoleucine, leucine and valine, are key both for protein synthesis and increased energy demand [70], so metastases must obtain them from circulation or surrounding tissues. Glutathione is a tripeptide of cysteine, glycine and glutamic acid that is an important antioxidant, and the ratio of reduced glutathione to oxidized glutathione within cells is a measure of cellular oxidative stress. Glutathione upregulation is associated with chemoresistance [71]. In the case of lipid metabolic changes, fatty acid synthase (FASN) promotes breast cancer metastasis and resistance development [72]. Interestingly, glucose metabolism links lipid and amino acid metabolism through the production of acetyl-CoA, which is key molecule involved in the oxidative metabolism of fatty acids and certain amino acids as part of the TCA cycle [68]. When glutamine levels are low, pyruvate carboxylase, a regulatory enzyme of gluconeogenesis, can allow fibroblasts to use extracellular lactate to maintain TCA cycle anaplerosis, non-essential amino acid biosynthesis, and extracellular matrix collagen production in the tumor microenvironment [73].

One of the smallest metabolites is nitric oxide (NO), which acts as a signal at low levels through cGMP and a defensive cytotoxin at high levels. Increased production of NO by iNOS dysregulates S-nitrosation to influence tumor initiation and metastasis. NO epigenetic effects are mediated by transcriptional regulation of histone-modifying enzymes and by perturbing their activities and cellular localizations via formation of iron–nitrosyl complexes and S-nitrosothiols. So iNOS inhibitors may be an unrecognized tool to reduce NO metabolite levels to control their epigenetic activity and metastasis [74].

## Epigenetic reprogramming of metabolic pathways – a root cause of cancer etiology and progression

Epigenetic modulators act during tumor progression to impose restrictions on the expression of tumor suppressor genes while inducing the expression of oncogenes. Epigenetic changes such as DNA methylation and the covalent modification of histone and non-histone proteins work in concert to augment metabolic adaptations during tumorigenesis as well as metastasis. Histone modifications lead to changes in chromatin architecture, that in turn regulate transcription of metabolic genes in response to various extra- and intracellular cues [75]. Remarkably, epigenetic regulation modulates the expression and function of different oncogenic signaling cascades that promote metabolic pathways in cancer. Thus, metabolomics and epigenomics are advancing together as prominent molecular and analytical methodologies for biomarker identification. DNA methylation processes are influenced by methionine and folate cycle metabolites. Altered concentrations of TCA cycle intermediates, including α-ketoglutarate (α-KG), succinate, fumarate, and acetyl-CoA metabolites, impact histone demethylases and enzymes catalyzing hydroxylation and demethylation processes, to thereby shape the cancer epigenetic landscape [76].

### Epigenetic regulation of glucose metabolism

**Fig. 1:**
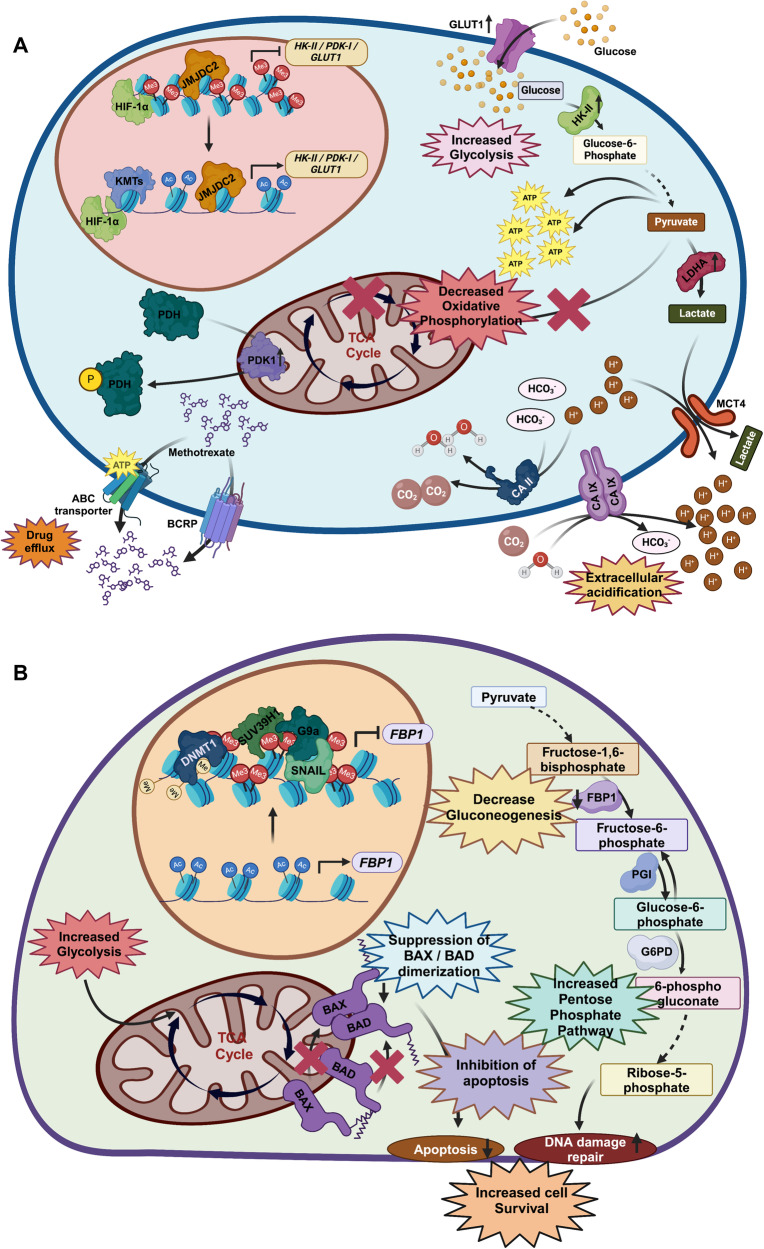
Epigenetic regulation on glycolytic and gluconeogenic genes contributes to the development of drug resistance. **A** HIF1α mediated epigenetic regulation causes active transcription of different glycolytic genes, thereby empowering ATP-dependent drug efflux pumps and inhibiting basic drug import. **B** Epigenetic repression on gluconeogenic genes and increased glycolysis cause inhibition of apoptosis. Glycolytic intermediates contribute to the pentose phosphate pathway which renders enhanced DNA damage repair.

In different cancers, the expression of key glucose uptake and glycolytic genes is augmented by various epigenetic regulations. For instance, hypermethylation of Derlin-3 promoter, which causes proteasomal degradation of GLUT1, leads to high glucose uptake and increased aerobic glycolysis [77]. Interestingly, most glycolytic genes have hypoxia-response elements (HREs) upstream of their promoters where HIF1α gets recruited upon induction of hypoxia to upregulate those genes. Evidence suggests that the Jumonji-C-histone demethylase family member JMJD2C acts as a transcription coactivator to selectively interact with HIF1α and remove H3K9 trimethylation marks at the HREs to augment HIF1α occupancy on its target gene promoters (Fig. 1A) [78]. This leads to upregulation of metabolic enzymes (such as GLUT1, HKII, PKM2, and LDHA expression) supporting metastasis of TNBC [78]. Also, hypomethylation of the pyruvate kinase M2 (PKM2) promoter is associated with increased expression in multiple cancers [79]. PKM2 connects glycolysis with other biosynthetic pathways of macromolecule production which in turn support enhanced proliferation of cancer cells [80]. Moreover, NAD-dependent deacetylase sirtuin 2 (SIRT2), which suppress neural-precursor-cell-expressed developmentally down-regulated (NEDD4) expression to inhibit c-Myc degradation [81], deacetylates PKM2 under nutrient-deprived condition. This allows formation of an active PKM2 tetrameric enzyme and conversion of phosphoenolpyruvate and ADP to pyruvate and ATP. The accumulated pyruvate then feeds the oxidative phosphorylation (OXPHOS) pathway. High OXPHOS promotes tumor proliferation-permissive resistant phenotypes. Yet, under excess nutrient condition, low SIRT2 activity increases PKM2 acetylation and enzymatic function, thereby triggering lactate production and the Warburg effect [82]. Ozden and his colleagues reported that SIRT3 deacetylates the Pyruvate Dehydrogenase E1 Subunit Alpha 1 (PDHA1) subunit of pyruvate dehydrogenase complex (PDC) at the K321 position, thereby increasing its activity, which reverses the Warburg effect as glucose consumption and lactate production are reduced [83].

In breast cancer, expression of SIRT3 at both the mRNA and protein levels is low [84]. Moreover, in breast carcinoma, SIRT6 acts as a tumor suppressor [85], which is correlated with an increased H3K9ac mark resulting in enhanced expression of glycolytic genes [86]. SIRT7 promotes isocitrate dehydrogenase 1 (IDH1) transcription in a SERPINE1 mRNA binding protein 1 (SERBP1) dependent manner to increase the α-keto-glutarate level and thereby enhance gluconeogenesis and lipogenesis, and destabilize HIF1α, to reverses glycolysis [87]. Basically, this SIRT7–IDH1 axis works to regulate metabolic reprogramming in cancer cells and, hence can help with therapeutic interventions [87]. Apart from epigenetic enzymes, the roles of different chromatin readers, which regulate metabolic programs, have been investigated. For example, the histone reader tripartite motif-containing 24 (TRIM24), which recognizes both H3K4me3 and H3K23ac marks [88], upregulates both glycolysis and TCA cycle genes and is associated with the malignant transformation of normal mammary epithelial cells [89].

In breast cancer cells, DNA methylation-mediated silencing has been reported on the promoter regions of fructose-1,6-bisphosphatase 1 (FBP1) and FBP2, the rate-liming enzymes of gluconeogenesis [79]. Loss of FBP1 is related to DNA methylation by DNMT1 along with an increased level of H3K9me2 by G9a on its promoter, which forms a ternary complex with Snail during induction of EMT in basal-like breast cancer (Fig. 1B) [58]. Histone de-methylases, like lysine-specific demethylase 1 (LSD1), remove the H3K4me2 mark from the transcription start site of FBP1 and glucose-6-phosphatase (G6Pase) [90]. LSD1 overexpression is a predictive biomarker for ER-negative breast cancer [91]. Phenotypically, high LSD1-expressing breast cancers are highly proliferative, metastatic, and invasive [92], reflecting the loss of this regulatory mechanism.

### Epigenetic regulation of amino acid metabolism

The primary epigenetic regulation occurs through methylation of DNA and Histones, both of which require the essential amino acid methionine. Also, acetylation of Histones requires the acetyl group derived from acetyl-CoA, which is generated from the breakdown of fatty acids and amino acids such as valine, leucine, isoleucine, and lysine. The synthesis of methionine is carried out by methionine synthase that is subsequently converted to the universal methyl donor S-adenosyl methionine (SAM) by methionine adenosyl transferase (MAT). Importantly, SAM is then utilized for DNA and Histone methylation through specific methyl transferases leading to the generation of S-adenosyl-homocysteine (SAH). The SAM/SAH ratio plays an important role in the regulation of the global epigenetic processes and is limited by the availability of methionine [93, 94]. In cancer cells, MATs can be upregulated in order to increase the production of SAM and thus can be a target to enhance chemosensitivity of drugs such as cisplatin [95].

### Epigenetic regulation of lipid metabolism

Rapidly proliferating tumor cells require large amounts of lipids to make cell membrane and signaling lipids, and thus have hyperactive lipogenesis through the activation of genes such as FASN, which utilizes acetyl-CoA as a substrate [96]. Acetyl-CoA is also used in the mevalonate pathway that leads to tube production of cholesterol, which is essential for the production of various lipid hormones, such as estrogen, and various other lipoproteins. Because of the diverse role acetyl-CoA plays in linking the glucose, amino acid, and fatty acid metabolism, its level is tightly regulated in the cell by the PI3K-AKT-mTOR pathway. This regulation is essential for proper Histone acetylation which result in altered gene expressions [97].

Generally speaking, metabolites such as SAM, acetyl-CoA, and ATP are intricately linked to the histone modifications, such as methylation, acetylation and phosphorylation, respectively, and are to be considered contextually.

## Cancer cell metabolic gene pool in the regulation of drug resistance

Resistance to different cancer therapies, either intrinsic or acquired, engages several well-known anti-cancer drug resistance mechanisms, which are influenced by rewired carbohydrate metabolism in cancer [98]. The mechanisms underlying the acquisition of drug resistance include enhanced drug efflux, alteration of drug target, increased DNA damage repair, high proliferation and inhibiting apoptosis, etc.

### Glucose metabolism in drug resistance

Transmembrane transporters for drug efflux belong to ATP binding cassette (ABC) transporter superfamily. These ABC transporters require a continuous ATP supply in cancer cells. This intracellular ATP level is much higher in cancer than in normal cells due to heightened glucose uptake and glycolysis which is known as the Warburg effect [99]. Furthermore, the intracellular ATP level is even higher in therapy-resistant cancer cells as compared to its parental drug-sensitive cell line [99, 100]. Besides the increased glycolysis-driven ATP elevation, breast cancer cells depend on mitochondria-derived ATP (OXPHOS pathway) for fueling ABC transporters to efflux doxorubicin to mediate multi-drug resistance (MDR) phenotype (Fig. 1A) [101, 102].

The elevated energy and biomass demand in drug-resistant cells is consistent with augmented drug efflux and detoxification mechanisms [103]. These demands are supported by concomitant changes in the metabolic milieu of cancer cells. Enhanced aerobic glycolysis is intricately connected to the development of resistance properties in breast cancer cells. So, abnormal expression and function of different glycolytic enzymes, contribute to drug resistance in cancer. Proviral insertion in Murine lymphomas (PIM2), a serine/threonine kinase and a proto-oncogene [104], phosphorylates hexokinase II (HKII), which results in increased enzyme stability and activity [105]. Both PIM2 and HKII expression is high in breast cancer cells, resulting in enhanced glycolysis and cellular growth, which has been associated with paclitaxel resistance [105]. 6-phosphofructo-2-kinase/fructose-2,6-biphosphatase 3 (PFKFB3), a vital regulator of glycolysis, is also controlled by HER2 signaling and has important role in HER2-positive breast cancers [106]. Employing PFKFB3 inhibitor in combination with HER2 inhibitor trastuzumab could be effective in sensitizing resistant breast cancer.

Enolase (EN), a key glycolytic enzyme is overexpressed in ER+ breast cancer patient samples [107]. Silencing EN promotes cytotoxicity to tamoxifen in the treatment of tamoxifen-resistant breast cancer cells [108]. Augmented expression of PKM2 in advanced breast carcinoma is correlated with cisplatin resistance [109]. Moreover, high expression of PKM2 in ER+ breast cancer models, MCF7 and T47D cell lines, heightens aerobic glycolysis and confers chemotherapy resistance [110]. In Trastuzumab-resistant ErbB2-positive breast cancer, inhibition of dysregulated glycolysis by 2-deoxy-D-glucose (2-DG) or the LDHA inhibitor oxamate promotes therapeutic efficacy in combination with trastuzumab. In paclitaxel-resistant TNBC cell lines, LDHA inhibitor re-sensitizes cells to paclitaxel [11]. Interestingly, excess lactate production remodels the tumor microenvironment by promoting acidosis, which leads to immune-suppression and therapeutic resistance [111].

There is an exchange of lactate between two different regions within the tumor microenvironment – the hypoxic region and the normoxic region. For instance, lactate uptake occurs in aerobic regions of breast cancer [112] which possess high expression of the monocarboxylate transporter MCT1 [113]. MCT4 acts in the release of lactate from hypoxic cancer cells [113]. Another pH regulator, carbonic anhydrase IX, is overexpressed on the plasma membrane when exposed to an extracellular acidic microenvironment in breast cancer, and it inhibits the import of basic drugs [114]. Under acidic conditions, electrostatic interactions between Breast Cancer Resistant Protein (BCRP) and methotrexate increase, which mediates drug efflux, reducing cytotoxicity of methotrexate [115]. Pyruvate dehydrogenase, the enzyme that facilitates the conversion of pyruvate to acetyl-CoA in the TCA cycle, gets phosphorylated and inhibited by pyruvate dehydrogenase kinase (PDK), further driving cellular metabolic pathways more towards glycolysis. PDK4 promotes anti-estrogen resistance in breast cancer [116].

Cancer cells show remarkable proficiency in DNA damage repair, which is a crucial mode of development of therapy resistance (Fig. 1B). As glycolytic intermediates contribute to the pentose phosphate pathway, it provides a nucleotide pool for DNA damage repair, augmenting resistance. Besides this, in chemoresistant breast cancer cells, the expression and activity of the aldehyde dehydrogenase (ALDH) enzyme are quite high which is an important detoxifying enzyme of glycolysis and combats oxidative stress [117]. Interestingly, aerobic glycolysis suppresses apoptosis induction, therefore conferring chemoresistance [113]. Mitochondrial metabolites (such as succinate, fumarate, and 2-hydroxyglutarate) are quantitatively elevated noticeably in tumors compared with normal cells. These dysregulate cellular processes including resistance by several mechanisms such as post-translational modifications of proteins and epigenetic regulations [118, 119]. In this context, succinate is known to stabilize HIF1α which induces chemoresistance through a pleiotropic mechanism [120]. Moreover, hyper-succinylation of nuclear factor erythroid 2-related factor 2 (NRF2) mediates a multi-drug-resistant (MDR) phenotype by upregulating resistance protein MRP1 and other drug-inactivating enzymes [121].

Thus, carbohydrate metabolism plays a seminal role towards the development of drug resistance in breast cancer and hence could be a potential target to sensitize the cancer cells towards the therapeutic regimen.

### Amino acid metabolism in drug resistance

With increased demand for nutrients during chemotherapy, cancer cells have been shown to upregulate L-type amino acid transporter 1 (also known as SLC7A5), which helps in the transportation of branched chain amino acids, such as leucine, isoleucine, valine, and bulky amino acids such as phenylalanine, tryptophan, tyrosine, methionine, glutamine, asparagine, and histidine, and is associated with TNBC [31, 122, 123]. Other amino acid transports such as SLC1A5 (for alanine, serine, and cysteine), and SLC7A11 (for cysteine) have been shown to be upregulated in TNBC and breast cancer therapy resistance [124, 125].

**Fig. 2:**
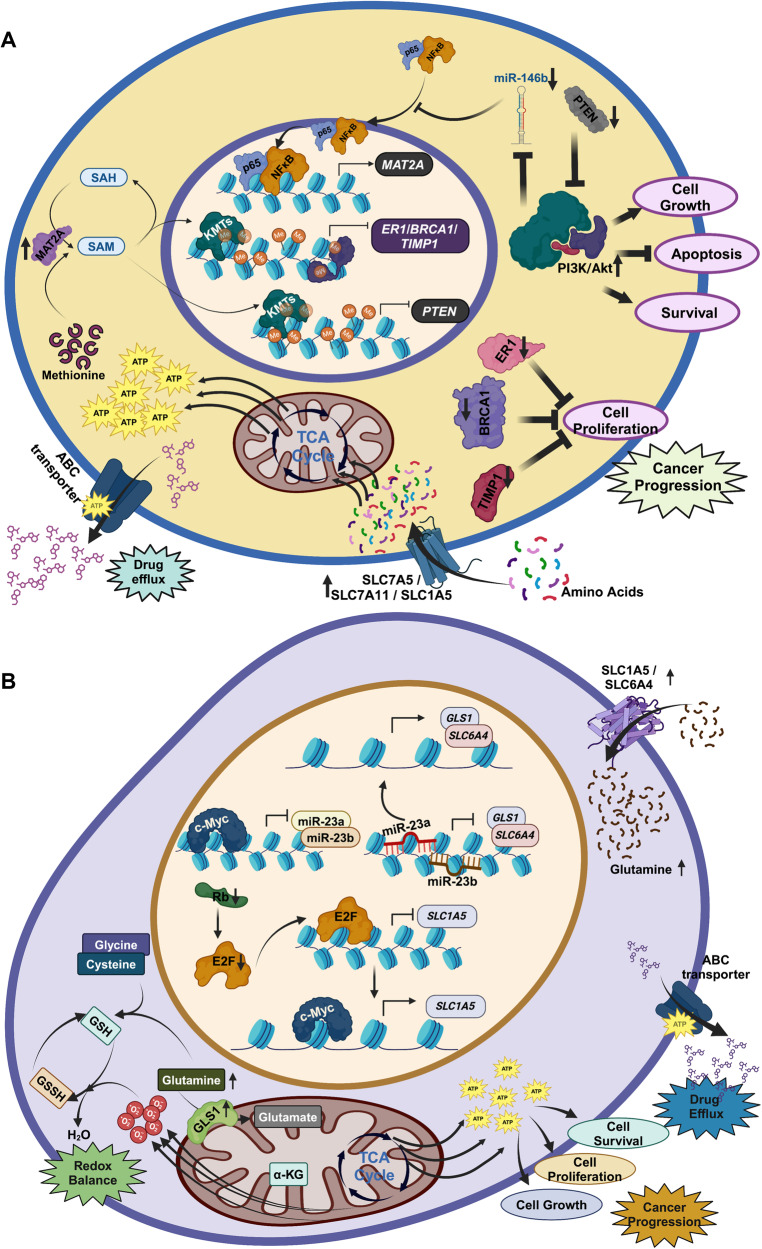
Epigenetic regulation of amino acid metabolic genes and its implication for therapeutic resistance in breast cancer. **A** Regulation of methionine metabolism and its role in drug efflux pump activation and cancer cell survival. **B** Epigenetic regulation of different amino acid transporter genes and glutamine metabolic genes and their enrollment in glutamine metabolism mediated adaptation in chemoresistance.

Exemplary important amino acids implicated in breast cancer and drug resistance are described here. Among them, Methionine acts as a substrate for one Carbon metabolism that supplies methyl group for histone methylation and DNA methylation. Upon resistance condition, p65 and NF-κB complex translocate inside the nucleus and upregulate the transcriptional expression of MAT2A [126], the enzyme responsible for the production of SAM from methionine [127, 128]. Then, different lysine methyl transferase (KMTs) and DNA methyl transferase (DNMTs) utilize SAM to methylate histones and DNA respectively to suppress the expression of different tumor suppressor proteins like ER1, BRCA1, and TIMP1 [127, 129]. Tumor suppressor gene PTEN, responsible for the suppression of PI3K/Akt pathway, gets hypermethylated by DNMT3a and become transcriptionally suppressed resulting in downregulation of PI3K/Akt pathway and thus suppress apoptosis and promote cell growth and proliferation [130, 131]. PI3K/Akt is also reported to suppress microRNA miR-146b that is responsible for the prevention of nuclear translocation of p65/NF-κB complex [132, 133]. Hence the upregulation of PI3K/Akt promotes this translocation and ultimately MAT2A transcription that leads to the formation of SAM from methionine to promote histone and DNA methylation (Fig. 2A). Finally, the essential amino acid methionine, has been shown to drive metastasis of TNBCs in vitro and in vivo and strategies towards its restriction could be used as a possible adjuvant therapy in TNBCs [134].

Glutamine supports cancer growth by acting as a nitrogen substrate for other amino acid synthesis, providing carbon source for the TCA cycle and fatty acid metabolism, and maintaining redox balance through glutathione [135]. Not surprisingly enzymes involved in glutamine metabolism have been shown to be upregulated in TNBC [136]. c-Myc binds to the promoters of miR-23a and miR-23b and suppresses its expression, thus activating GLS1 and SLC6A4 [137]. SLC6A4 increase the uptake of glutamine and GLS1 converts glutamine to glutamate and ultimately forms some intermediates of the TCA cycle [138]. A higher rate of the TCA cycle generates a higher amount of energy to support the function of the drug efflux pump and to promote cell growth and proliferation (Fig. 2B). Glutamine can also be converted into glutathione (GSH) and maintain the redox balance of the cell [139].

Notably amino acid transporters can transport both Methionine and Glutamine like SLC1A5, whose expression is also regulated by c-Myc in TNBC. E2F-3 is well known to suppress the expression of SLC1A5 [136]. During chemoresistance conditions, Rb protein levels decrease, leading to the overall reduction of E2F resulting c-Myc mediated activation of SLC1A5 and the rise of glutamine and methionine pool inside the cells [137, 140]. Glutamine can also act as a signaling molecule and activates mTORC1 signaling pathway resulting in cancer cell proliferation and suppression of autophagy-mediated cell death [141]. Suppression of glutamine transporter SLC1A5 leads to the downregulation of mTORC1 signaling resulting in autophagy-mediated cell death [142, 143]. A variant of SLC1A5 is reported to transport glutamine inside mitochondria. Overexpression of variant SLC1A5 lead to the overproduction of glutathione and cause gemcitabine resistance in pancreatic cancer [144].

In cancer cells, serine, a non-essential amino acid, drives growth by providing a one-carbon pool [145]. De novo synthesis of serine is dependent upon phosphoglycerate dehydrogenase (PHGDH), phosphoserine aminotransferase 1 (PSAT1) and phosphoserine phosphatase [146], the levels of which are upregulated in various cancers including breast [93, 147]. In conditions of glutamine depletion or mitochondrial dysfunction, cancer cells survive primarily through asparagine metabolism [148]. Moreover, tumor asparagine level has been strongly correlated with EMT and metastasis in breast cancers [149].

Collectively, amino acid metabolism plays a key role in the context of therapeutic resistance in breast cancer. Hence targeting a specific amino acid metabolism could be a strategy to alleviate the challenges of chemoresistance.

### Lipid metabolism in drug resistance

**Fig. 3:**
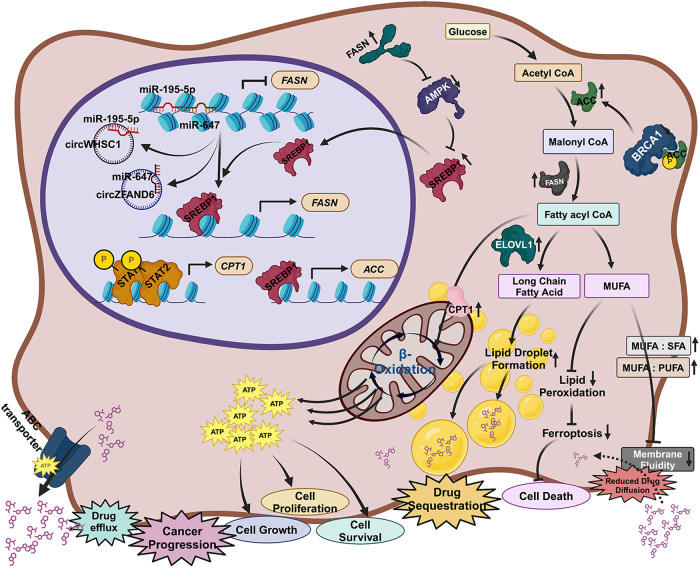
Epigenetic regulation of key lipid metabolic genes and its role in metabolic adaptation against therapeutic intervention in breast cancer. An epigenetic perspective of different lipid metabolic gene regulation and its implication for reducing the effect of chemotherapy and its role in cancer cell survival and proliferation.

Alterations in lipid metabolism play a major role in breast cancer development and progression. To meet the increased lipid needs, the rapidly growing tumor cells employ several processes such as increased uptake of extracellular lipids, increased de novo lipogenesis (the utilization of glucose or amino acids such as glutamine to make new fatty acids), and increased cellular storage lipid-droplets. Moreover, the mevalonate pathway generates cholesterol from acetyl-CoA or from low-density lipoprotein receptor (LDLR) acquired cholesterol [26]. Not surprisingly, enzymes that play a role in these processes have been implicated in breast cancer progression and chemoresistance (Fig. 3). For example, FASN activity has been associated with HER2 marker, metastasis, relapse and chemoresistance [72, 150]. The upregulation of FASN gene expression has been well documented in other subtypes of breast cancer including TNBC and was initially considered as a potential drug target [72]. In normal condition, miR-195-5p and miR647 binds to the promoter of FASN genes and suppress its activity [151]. Upon induction of therapeutic resistance in TNBC, the expression of circular RNA circWHSC1 and circZFAND6 increased resulting in sponging of miR-195-5p and miR647 respectively [152, 153]. Then SREBP1 binds to the promoter of FASN and activates its transcription [154]. Sterol regulatory element binding protein-1 (SREBP1) protein level increases when AMPK is suppressed by higher availability of FASN thus the FASN expression increases further in a positive feedback manner [155, 156]. The tumor suppressor protein BRCA1 is observed to bind with phosphorylated Acetyl-CoA carboxylase (ACC) to keep it stable in its functionally inactive form [157]. At chemoresistance, BRCA1 level decreases [158] resulting in dephosphorylation-mediated activation of ACC and increasing the production of malonyl CoA from Acetyl-CoA [159]. Then FASN converts the malonyl CoA into fatty acyl CoA [160]. Other two important genes studied to be activated upon resistant conditions are ELOVL [161] and CPT1 [162]. ELOVL promotes lipid droplet formation by synthesizing long-chain fatty acids, that sequester chemotherapeutic drugs [163]. On the other hand, CPT1 promotes β-oxidation mediated degradation of fatty acyl CoA and generates energy to support cell growth, cell proliferation, and drug efflux pump activation [164]. The increased amount of fatty acyl CoA also increases the ratio of monounsaturated fatty acid (MUFA) and saturated fatty acid (SFA) and also the ratio of MUFA and polyunsaturated fatty acids (PUFA) in the plasma membrane thus increase the membrane rigidity that prevent the diffusion of chemotherapeutic drugs through the plasma membrane [165]. Reduction of PUFA minimizes the lipid peroxidation-mediated ferroptosis thus suppress cell death [166].

Cancer-induced reprogramming of the sterol regulatory element binding protein-2 (SREBP-2) activated genes involved in the cholesterol biosynthesis pathway including those of the mevalonate pathway such as HMG-CoA reductase, mevalonate kinase, squalene synthase, etc, as well as increases expression of LDLR to acquire extracellular cholesterol [167].

Comprehensively, lipid metabolism is a potential regulatory circuit that can be targeted during therapeutic resistance in breast cancer. Mechanistically, targeting lipid metabolism can suppress cell proliferation and increase bio-availability of the chemotherapeutic agents.

## Overcoming therapy resistance by targeting the epigenome

Epigenetic regulators play integral roles in tumor initiation, progression, metastasis, drug resistance, and relapse [168–170]. Therefore, these epigenetic modifiers have been targeted for drug development for several years [171–173]. Recently, targeted reprogramming of the epigenetic milieu has evolved as a potential therapeutic approach. A number of epidrugs are developed that target the most crucial epigenetic regulators such as DNA methyltransferases and histone modifiers [174].

## Epigenetic therapy modulates metabolic programs

Metabolic reprogramming and epigenetic modifications play pivotal roles in tumor progression and in tumor microenvironment. Epigenetic drugs like DNA methyl transferase inhibitor (DNMTi) (5-Aza-2-deoxycytidines) and Histone deacetylase inhibitor (HDACi) (Belinostat, Vorinostat/SAHA, Romidepsin, and Panobinostat) are being used in combination with immunotherapy/ chemotherapy/targeted therapy for better efficacy in refractory/ resistant/relapsed breast tumors [172, 175, 176]. For instance, 5-Aza-2-deoxycytidine sensitizes doxorubicin-resistant breast cancer by reinstating MutS homolog 2 (MSH2) [177]. Interestingly, in doxorubicin-resistant tumors, inhibition of DNMTs also suppresses H3K27me3 and the Histone methyl transferase (HMT) associated with it, inhibiting global gene repression [177]. HDACis alone or in combination with DNMTis also resensitize the chemoresistant tumors [178]. HDACi resensitize the refractory cells by inducing apoptosis and autophagy and increasing DNA damage and ROS production. HDAC inhibitors, like butyrate and TSA, also reverse the dependency of breast cancer cells from aerobic glycolysis to oxidative phosphorylation [179]. Thus, HDACi are used in combination with DNMTi as better therapeutics for refractory tumors.

First-generation inhibitors (DNMTi and HDACi) are being given in combination with second and third-generation Histone methyl transferase inhibitor (HMTi), Histone acetyl transferase inhibitor (HATi), Histone demethylase inhibitor (HDMi), Bromodomain and extra terminal domain inhibitor (BETi), and Aurora kinase inhibitors [173]. These Epi-Drugs not only target epigenetic modifiers but also target cofactors like acetyl-CoA and S-Adenosyl methionine (SAM), which are also chief oncometabolites. SAM availability is crucial for both DNMT and HMT activity in inducing resMDR1istant genes and repressing tumor suppressor expression. Therefore, targeting the DNMTs by 5-Aza, along with SAM inhibitors, shows potency in hindering the metabolic adaptations of resistant tumors. In resistant tumors with the altered metabolic landscape, these epi-drugs are used in combination with other metabolic drugs including antimetabolites, mTOR/kinase inhibitors, PARP inhibitors, retinoids, deaminase inhibitors alkylating agents, and taxanes [173].

The cofactors involved in histone acetylation such as acetyl-CoA and citrate promote glycolytic flux in tumors [180]. Therefore, 2-DG treatment, which competitively inhibits glucose-6-phosphate (G6P) production, suppresses acetyl-CoA levels, thereby sensitizing cells to chemotherapeutic drugs [181]. Furthermore, Sirtuins, specifically SIRT6, have crucial roles in the metabolic regulation of resistant cancer. Nicotinamide, a well-known sirtuin inhibitor, thus assists in targeting cancer cell metabolism [182]. Several preclinical studies have therefore highlighted the potency of targeted epigenetic reprogramming in sensitizing refractory tumors to therapy by inducing apoptosis, inhibiting EMT and stemness, regulating TME, and modulating cancer cell metabolism [174]. An as yet unrealized opportunity will be jointly employing epigenetic inhibitors with compounds that target metabolic alterations that can synergistically impact epigenetic regulation by modulating the availability of metabolites acting in epigenetic modifications.

## Epigenetic rewiring to combat drug resistance

Dynamic and reversible changes in the epigenome, provide new avenues for therapeutic regimes via re-sensitization of resistant tumor cells [183]. Epigenetic mechanisms of resensitization are highly dynamic due to the large degree of tissue heterogeneity within resistant tumors. Recent studies highlight the contribution of epigenetic modifiers in acquired drug resistance. For instance, DNMTs, HDACs, HMTs, and HDMs (like lysine specific protein demethylase KDM2/3/5/6/7) play crucial roles in inducing drug-resistance, concomitantly with the induction of stem cell-like features [184–187]. Accumulating evidence suggests that inhibitors targeting epigenetic modifiers in combination with chemotherapeutic drugs help to re-sensitize chemoresistant cells [177, 188]. Various solid tumors including breast cancer increase DNA damage-mediated apoptosis when treated with the HDAC inhibitor Suberoylanilide Hydroxamic Acid (SAHA) in combination with the chemotherapeutic drug cisplatin [189]. SAHA treatment reverses the silencing of pro-apoptotic gene protease-activated receptor-4 (PAR-4) by rescuing its promoter from HDAC-mediated deacetylation, ultimately leading to sensitization of recurrent breast tumor cells to cytotoxic chemotherapy. Despite the success in combination therapy, SAHA is not mainstream due to inconsistencies in large scale clinical trials results, adverse side effects, and increased costs [190].

Enhancer of zeste homolog 2 (EZH2), lysine demethylase 6B (KDM6B), and bromodomain-containing proteins are responsible for inducing resistant cells via changing the epigenetic landscape and remodeling the chromatin architecture [186]. Thus, BET inhibitors re-sensitize cells to drug treatment by blocking the interaction of BRD4 with YAP/TAZ [191]. Activation of the PI3K pathway and transcriptional reprogramming due to resistance to BET inhibition promotes the BRD4/CDK6/FOXO3a axis in luminal breast cancers [192]. Thus, inhibitors of epigenetic modifiers, at low doses, activate tumor suppressors and induce differentiation of CSCs to prevent invasion and metastasis [183].

Epigenetic deregulation of drug efflux transporters (like multidrug resistance protein 1 MDR1, MRP1/2, and BCRP) is correlated with acquired chemoresistance [193, 194]. For instance, hypomethylation of the MDR1 promoter, leading to elevated expression, is reported to aid in the acquisition of therapy resistance in breast cancer [195]. So, targeting these deregulatory mechanisms may re-sensitize these cells towards conventional therapeutic strategies. Therefore, epigenetic repression of drug efflux pumps may sensitize resistant cancer cells to different chemotherapeutic agents [196, 197].

These findings imply that reprogramming the epigenetic landscape with epigenetic modifiers alone or in combination with other drugs is a logical therapeutic strategy against advanced forms of breast cancer.

The combinatorial therapy with epi-drugs regulating both DNA methylation and histone modifications may also serve as a promising therapeutic approach. For instance, in Tamoxifen-resistant breast cancer cells, ER gene remains repressed through both promoter methylation and histone deacetylation [198]. Thus, co-treatment with HDAC and DNMT inhibitors may display significant potential in restoring tamoxifen sensitivity [199]. However, a thorough investigation of the central cellular epigenetic regulatory networks is necessary for developing potent anti-cancer epi-therapeutic strategies with low off-target effects [200].

## Clinical trials of epi-drugs for drug-resistant breast cancer

The epigenome and its regulators play intricate roles in cellular homeostasis and survival. Therefore, therapeutic approaches using epigenome-modulating drugs may cause systemic toxicity. Due to this scenario, clinical trials of epi-drugs continue to be required to optimize therapeutic doses and minimize systemic toxicity. So, several epi-drugs are under ongoing clinical trials in resistant breast cancer, alone or in combination with other endocrine or chemotherapeutic drugs (https://www.clinicaltrials.gov/).

The class I HDAC inhibitor romidepsin (FK228) has completed a phase II clinical trial in metastatic breast cancer (NCT00098397). In HER2 locally recurrent or metastatic breast cancer, a phase II clinical trial was completed to evaluate the effect of pan-HDAC inhibitor Panobinostat (NCT00777049). The pan-HDAC inhibitor valproic acid has been under several clinical trials for the treatment of breast cancer. A phase II clinical study (NCT01010854, currently terminated) utilized valproic acid along with FEC (5-fluorouracil, epirubicin, and cyclophosphamide) in patients with primary or locally advanced metastatic breast carcinoma. Another clinical trial (NCT00395655) reported the utilization of demethylating agent hydralazine along with valproic acid plus neoadjuvant cyclophosphamide and doxorubicin therapy in locally advanced breast cancer patients. An ongoing clinical trial (NCT01552434) in patients with advanced metastatic and/or recurrent breast cancer is reportedly utilizing valproic acid as a combinatorial drug along with bevacizumab and temsirolimus.

The HDAC inhibitor entinostat has been used in co-treatment with other drugs in several clinical trials for breast cancer patients. In a phase II clinical trial in postmenopausal women with TNBC, entinostat was used in combination with the aromatase inhibitor anastrozole (NCT01234532). Another aromatase inhibitor exemestane has been used in combination with entinostat in several breast cancer clinical trials (NCT02115282, NCT02833155, NCT02820961, NCT00676663, NCT03538171, NCT03291886, NCT02623751, NCT03280563). Entinostat has also been used in several clinical trials in combination with different monoclonal antibodies like anti-PDL1 antibody atezolizumab (NCT02708680, Phase II), anti-VEGF antibody bevacizumab (NCT03280563, Phase II, recruiting) and anti-CTLA4 antibody ipilimumab (NCT02453620, Phase I). In ER+ breast cancer patients, entinostat has been evaluated in a clinical trial as a combinatorial therapy with ER inhibitors fulvestrant and tamoxifen (NCT03280563, recruiting). The combination of entinostat with lapatinib ditosylate or trastuzumab has completed a Phase-I clinical trial (NCT01434303) showing safety and potential clinical benefit in treating relapsed metastatic breast cancer patients, who were previously treated with trastuzumab only [201].

In tamoxifen-resistant breast cancer models, the HDAC inhibitor vorinostat and tamoxifen synergistically reverted Bcl-2 expression and promote apoptosis [202]. Interestingly, this combination is being evaluated in a Phase-II actively recruiting clinical trial for the treatment of ER+ breast cancer patients (NCT04190056).

Small molecule inhibitors against BET proteins have also been used as epi-drugs in several clinical trials for breast cancer patients. For instance, molibresib or I-BET62, an orally bioavailable small-molecule BET inhibitor has been assessed in Phase I clinical trials in combination with the estrogen receptor inhibitor fulvestrant in ER+ breast cancer patients (NCT02964507). Another novel BET inhibitor RO6870810 or TEN-010 has been under Phase I clinical trial along with anti-PDL1 antibody atezolizumab in TNBC patients (NCT03292172). Recently, a Phase I clinical trial in ER+ breast cancer patients has assessed maximum tolerated dose of BET inhibitor Alobresib or GS-5829 as a combinatorial drug with exemestane and fulvestrant (NCT02392611).

The LSD1/KDM1A inhibitor phenelzine, which is a potent anti-depressant, was recently been repurposed in a Phase I clinical trial along with nanoparticle-bound paclitaxel in patients with advanced breast carcinoma (NCT03505528). The DNMT inhibitor azacytidine has been extensively utilized in hematological malignancies and has also been assessed in advanced or metastatic breast cancer patients. A Phase II clinical trial of azacytidine was completed in metastatic breast cancer patients in combination with nanoparticle albumin-bound paclitaxel (Abraxane) (NCT00748553). An ongoing Phase II clinical trial with azacytidine is currently in progress in high-risk early-stage breast cancer patients (NCT04891068). Another well-established DNMT inhibitor, decitabine, is also being evaluated along with chemotherapy in several clinical trials in breast cancer (NCT02957968, NCT03282825, NCT03295552).

These completed and ongoing clinical studies underscore the potential utility of epigenetic modulatory molecules as novel therapeutic strategies to complement the conventional breast cancer therapeutics.

## Future directions

Increasing evidence linking metabolic dysregulation to the acquisition of drug resistance in cancer cells, motivates emergence of new therapeutic regimens to re-sensitize and kill tumor cells via epi-metabolic targeting. Breast cancer treatment conventionally relies primarily on surgery, radio-, chemo- and endocrine therapies. However, acquisition of resistance towards chemotherapy as well as hormonal therapies, and subsequent tumor relapse, continue to pose huge challenges for breast cancer management.

In this context, diverse small molecules targeting cancer metabolism have been identified for breast cancer, particularly those targeting enzymes involved in glycolysis, glutaminolysis, and fatty acid synthesis. These therapies may serve to enhance the efficacy of current therapies and re-sensitize resistant cancer cells. Yet, to date, such approaches have not succeeded in later stage clinical trials. Probable reasons for such failures are the complexity of metabolic pathways, crosstalk among complex signaling pathways, and the potential systemic toxicity of metabolic drugs, as both cancer and normal cells depend on these metabolic pathways for energy production. Attempts at low dosing with these drugs to avoid toxicity may instead lead to gain in resistance and pose added challenges.

Interactions between onco-metabolic signaling and epigenetic machinery are relevant to accurate identification and understanding of the epigenetic state of specific cancer cells, thereby aiding their targeting for better therapeutic response. Decoding the dynamic epigenetic landscape at the level of metabolic molecular mechanisms will be crucial for enhancing the efficacy of Epi-Drugs, used in combination therapy. Importantly, due to tumor metabolic heterogeneity, the same epigenetic therapy cannot simply be assigned to all patients of a particular cancer type. Pre, post, and during treatment, genome-wide analysis followed by metabolic tumor profiling promises to assist in designing personalized epi-metabolic therapy and thereby minimize resistance and disease relapse. Yet, the important potential to synergistically target the two cancer hallmarks of altered metabolism and epigenetics will likely require improved and comprehensive dynamic network models. Successful models will capture the interwoven interactions of epigenetic regulation and altered metabolic activities plus their temporal and spatial interconnection with tumor microenvironment and other pathways including DNA damage responses. Ongoing advancements in these fields are anticipated to open new paths to development of successful mechanism-based epi-metabolic therapies as an essential component in advanced comprehensive and precision breast cancer treatment.
